# Synthesis, crystal structure and aggregation-induced emission of a new pyrene-based compound, 3,3-diphenyl-2-[4-(pyren-1-yl)phen­yl]acrylo­nitrile

**DOI:** 10.1107/S2056989018005182

**Published:** 2018-04-06

**Authors:** Bao-Xi Miao, Xin-Xue Tang, Li-Fang Zhang

**Affiliations:** a School of Chemical Engineering and Technology, China University of Mining and Technology, Xuzhou 221116, People’s Republic of China

**Keywords:** synthesis, aggregation-induced emission, pyrene, crystal structure

## Abstract

The title organic compound, C_37_H_23_N, crystallizing in the triclinic space group *P* , has been designed, synthesized and characterized by single-crystal X-ray diffaction, MS, NMR and elemental analysis. There are alternating relatively strong and weak intermolecular π–π interactions between adjacent pyrene ring systems, forming a one-dimensional supramolecular structure. The compound is weakly fluorescent in THF solution, but it is highly emissive in the condensed phase, revealing distinct aggregation-induced emission (AIE) characteristics.

## Abstract 2

There are alternating relatively strong and weak intermolecular π–π interactions between adjacent pyrene ring systems, forming a one-dimensional supramolecular structure. The compound is weakly fluorescent in THF solution, but it is highly emissive in the condensed phase, revealing distinct aggregation-induced emission (AIE) characteristics.

## Chemical context   

Over the last several decades, research on organic fluorescent materials has gained important momentum because of their wide range of applications in organic light-emitting diodes (OLED), organic field-effect transistors (OFET), organic lasers, fluorescent sensors and solar cells and so on (Indumathi *et al.*, 2017 ▸; Mishra *et al.*, 2011 ▸; Nie *et al.*, 2017 ▸; Sasabe *et al.*, 2011 ▸; Zhao *et al.*, 2010 ▸). As a well known fluoro­phore, pyrene and its derivatives have attracted much attention owing to its pure blue fluorescence with high quantum yield, exceptionally long fluorescence lifetime, excellent thermal stability and high charge-carrier mobility (Figueira-Duarte *et al.*, 2011 ▸; Luo *et al.*, 2001 ▸; Zhang *et al.*, 2016*d* ▸, 2017 ▸). However, pyrene-based compounds show notorious aggregation-caused quenching (ACQ), which severely limits their application range. Encouragingly, the discovery of aggregation-induced emission (AIE) by Tang and co-workers has opened up a new approach for excellent emission materials in the solid state (Yuan *et al.*, 2013 ▸). Indeed, propeller-like conformations such as tetra­phenyl­ethene (TPE) and tri­phenyl­acrylo­nitrile (TPAN) have been widely used for the design of AIE-active compounds because of their easy preparation and outstanding AIE effects (Han *et al.*, 2016 ▸; Jadhav *et al.*, 2015 ▸; Lu *et al.*, 2015 ▸; Tasso *et al.*, 2015 ▸; Zhang *et al.*, 2016*a* ▸). Compared to the propeller-shaped AIE-active moiety TPE, TPAN also exhibits typical crystallization-induced emission (CIE) behaviours, so the combination of TPAN with other fluoro­phores can readily generate mechanochromic materials, displaying reversible solid-state emission upon mechanical stimuli and solvent evaporation (Hirata *et al.*, 2006 ▸; Zhang *et al.*, 2016*b* ▸). As a result of their promising potential applications in optical recording and as fluorescent switches and security inks, these mechanochromic materials have attracted considerable attention (Srinivasan *et al.*, 2009 ▸; Zhang *et al.*, 2018 ▸). Herein, we report the synthesis and crystal structure of a new pyrene-based tri­phenyl­acrylo­nitrile, 2-[4-(1-pyren­yl)phen­yl]-3,3-di­phenyl­acrylo­nitrile, using a Suzuki cross-coupling reaction between 2-(4-bromo­phen­yl)-3,3-di­phenyl­acrylo­nitrile and 1-pyrenylboronic acid, which may exhibit both AIE and mechanochromic characteristics.
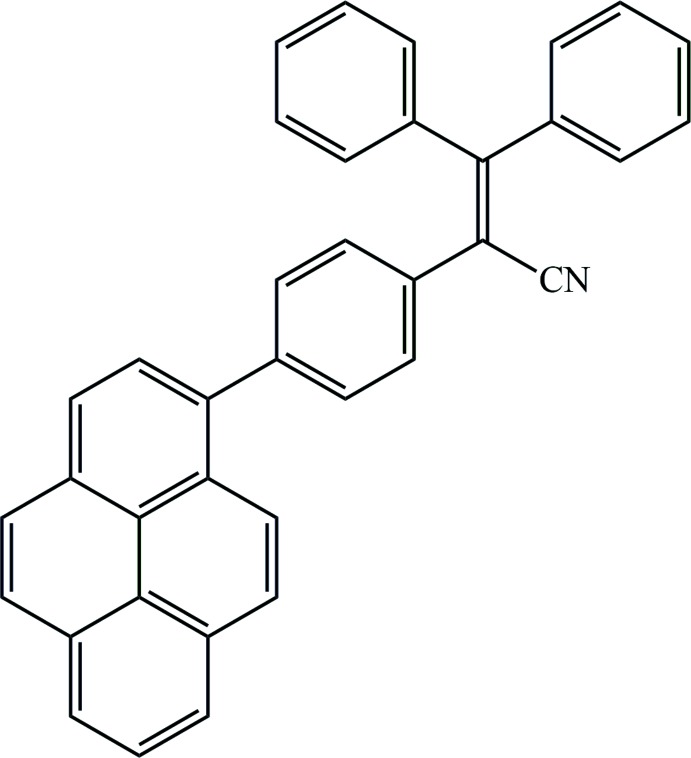


## Structural commentary   

**Figure 1:**
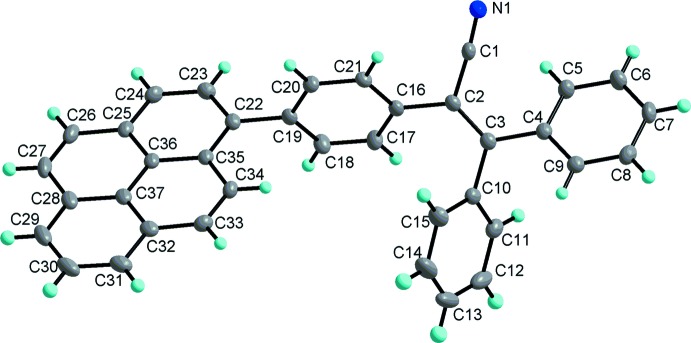
The mol­ecular structure of the title complex, with 30% probability displacement ellipsoids.

The single X-ray diffraction analysis agrees well with the expected structure of the title compound, as shown in Fig. 1 ▸. The 2,3,3-tri­phenyl­acrylo­nitrile unit, which exhibits the typical propeller-shaped structure, is linked by a planar pyrenyl unit at one phenyl segment. The length of the central C2—C3 bond is 1.3623 (14) Å, which is typical for a double C=C bond. The C—N bond length is 1.1479 (14) Å, which is comparable with those of other cyanide-containing organic or inorganic compounds, showing the existence of a cyanide group. The pyrenyl ring system is almost strictly planar, with the largest derivation from the mean plane being 0.027 (3) Å for atom C31.

## Supra­molecular features   

**Figure 2:**
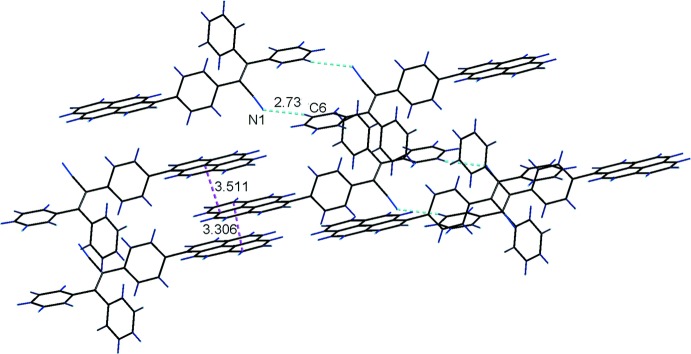
The supra­molecular structure of the title compound built up through π–π and C—H⋯N inter­actions.

In the crystal, there are alternating relatively strong and weak inter­molecular π–π inter­actions between adjacent pyrene ring systems with shortest inter­atomic distances C26⋯C37(1 − *x*, −*y*, 2 − *z*) = 3.511 (3) and C31⋯C31(2 − *x*, −*y*, 2 − *z*) = 3.306 (3) Å, which link the mol­ecules into a one-dimensional supra­molecular structure. In addition, there are C6—H6⋯N1 inter­actions with a C⋯N distance of 3.3563 (17) Å (Table 1 ▸) between the cyanide nitro­gen atom and a benzene carbon atom, which link the above one-dimensional supra­molecular structures into two-dimensional supra­molecular networks parallel to (010), as shown in Fig. 2 ▸. These inter­molecular inter­actions can be compared with those in 1-pyrenyl-based triaryl­amines (Zhang *et al.*, 2016*c* ▸).

## Aggregation-induced emission   

**Figure 3:**
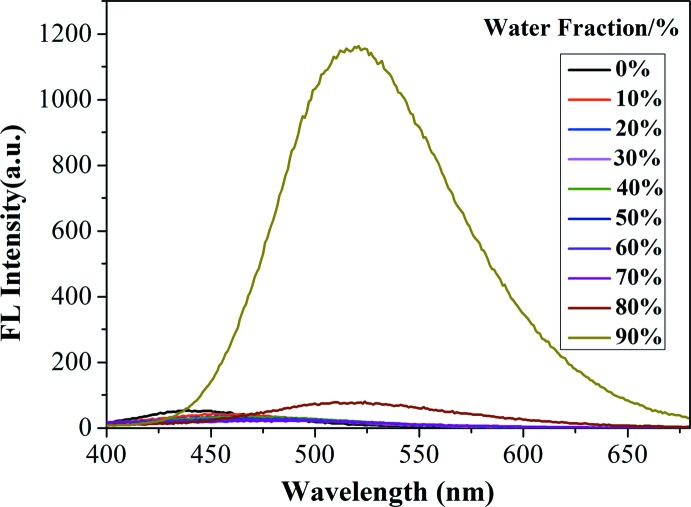
Fluorescence spectra of the title compound in water–THF mixtures with different water fractions.

The corresponding emission spectra of the title compound in aqueous THF with different water/THF ratios at a concentration of 5 × 10^−5^ *M* are shown in Fig. 3 ▸. It can be seen that the title compound shows weak fluorescence when the water fraction is below 70%, which is ascribed to the active intra­molecular rotations of the genuinely dissolved luminogens in these mixtures. The yellow fluorescence starts to increase gradually at a water content of 80%, at which the luminogens begin to aggregate, and reaches a maximum, which is nearly 50 times stronger than that in the pure THF solution, when the water content is 90%. The title compound therefore exhibits typical aggregation-induced emission (AIE) activity.

## Database Suvey   

The structure of the title compound can be compared with our previously reported seriors of pyrenyl-based triaryl­amines in which two compounds crystallize in the same *P* 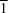 space group (Zhang *et al.*, 2016*c* ▸). In these compounds, the substituent groups are all at the 1-position of the pyrene ring system. Importantly, because of the existence of the relatively larger planar pyrene ring system, there are inter­molecular π–π inter­actions between adjacent pyrene ring systems, providing evidence that the presence of a pyrene ring system is favorable for the formation of strong inter­molecular inter­actions.

## Synthesis and crystallization   

The starting material 2-(4-bromo­phen­yl)-3,3-di­phenyl­acrylo­nitrile was synthesized according to the literature (Wang *et al.*, 2000 ▸). All other chemicals were purchased from commercial sources and used as received without further purification. A mixture of 2-(4-bromo­phen­yl)-3,3-di­phenyl­acrylo­nitrile (1.8013 g, 5 mmol), 1-pyrenylboronic acid (1.2304 g, 5 mmol), catalyst Pd(PPh_3_)_4_ (0.1156 g, 2 mol%), K_2_CO_3_ (2.7642 g, 20 mmol, dissolved in 5 mL of water) and 20 mL of MeOH in 80 mL of toluene was stirred at 353 K for 16 h. The reaction mixture was then cooled down and extracted with methyl­ene dichloride. The combined organic layer was dried over anhydrous MgSO_4_ and filtered. The solvent was removed and the residue was purified by silica gel chromatography using hexa­ne/methyl­ene dichloride (*v*/*v* = 1:1) as eluent to afford the title compound (2.0683 g; yield 86%). Light-yellow block-shaped crystals were obtained by slow evaporation of a hexa­ne/methyl­ene dichloride solution (*v*/*v* = 1:1)

^1^H NMR (600 MHz, chloro­form-*d*) δ 8.27–8.18 (*m*, 3H), 8.16–8.10 (*m*, 3H), 8.09–8.02 (*m*, 2H), 7.97 (*d*, *J* = 7.8 Hz, 1H), 7.59–7.46 (*m*, 9H), 7.40–7.29 (*m*, 3H), 7.21–7.15 (*m*, 2H). MALDI–TOF MS: *m*/*z* calculated for C_37_H_23_N 481.5853, found 481.5806 [*M*]^+^. Elemental analysis calculated for C_37_H_23_N: C, 92.18%; H, 4.86%; N, 2.85%; found: C, 92.28%, H, 4.81%; N, 2.91%.

## Refinement   

Crystal data, data collection and structure refinement details are summarized in Table 2 ▸. Hydrogen atoms were placed in calculated positions C—H = 0.93 Å) and refined using a riding model with *U*_iso_(H) = 1.2*U*_eq_(C).

## Supplementary Material

Crystal structure: contains datablock(s) I, 1. DOI: 10.1107/S2056989018005182/eb2006sup1.cif

Structure factors: contains datablock(s) I. DOI: 10.1107/S2056989018005182/eb2006Isup2.hkl

Supporting information file. DOI: 10.1107/S2056989018005182/eb2006Isup3.cml

CCDC reference: 1834096

Additional supporting information:  crystallographic information; 3D view; checkCIF report

## Figures and Tables

**Table 1:** Hydrogen-bond geometry (Å, °)

*D*—H⋯*A*	*D*—H	H⋯*A*	*D*⋯*A*	*D*—H⋯*A*
C6—H6⋯N1^i^	0.93	2.73	3.3563 (17)	125

Symmetry code: (i) 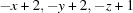.

**Table 2:** Experimental details

Crystal data	
Chemical formula	C_37_H_23_N
*M*_r_	481.51
Crystal system, space group	Triclinic, *P* 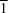
Temperature (K)	123
*a*, *b*, *c* (Å)	9.2277 (2), 10.6445 (3), 14.639 (2)
α, β, γ (°)	105.169 (2), 94.806 (2), 113.255 (2)
*V* (Å^3^)	1246.38 (18)
*Z*	2
Radiation type	Mo *K*α
μ (mm^−1^)	0.07
Crystal size (mm)	0.12 × 0.12 × 0.10

Data collection	
Diffractometer	Bruker APEXII CCD area-detector
Absorption correction	Multi-scan (*SADABS*; Sheldrick, 2015 ▸)
*T*_min_, *T*_max_	0.981, 0.995
No. of measured, independent and observed [*I* > 2σ(*I*)] reflections	22549, 5093, 4402
*R*_int_	0.026
(sin θ/λ)_max_ (Å^−1^)	0.625

Refinement	
*R*[*F* ^2^ > 2σ(*F* ^2^)], *wR*(*F* ^2^), *S*	0.035, 0.104, 1.04
No. of reflections	5093
No. of parameters	343
H-atom treatment	H-atom parameters constrained
Δρ_max_, Δρ_min_ (e Å^−3^)	0.22, −0.19

Computer programs: *APEX2* and *SAINT-Plus* (Bruker, 2001 ▸), *SHELXS2014* (Sheldrick, 2008 ▸), *SHELXL2014* (Sheldrick, 2015 ▸) and *DIAMOND* (Brandenburg, 2005 ▸).

## supplementary crystallographic information

### Crystal data

**Table:**

C_37_H_23_N	*Z* = 2
*M**_r_* = 481.51	*F*(000) = 504
Triclinic, *P*1	*D*_x_ = 1.283 Mg m^−^^3^
*a* = 9.2277 (2) Å	Mo *K*α radiation, λ = 0.71073 Å
*b* = 10.6445 (3) Å	Cell parameters from 2445 reflections
*c* = 14.639 (2) Å	θ = 3.0–26.4°
α = 105.169 (2)°	µ = 0.07 mm^−^^1^
β = 94.806 (2)°	*T* = 123 K
γ = 113.255 (2)°	Block, yellow
*V* = 1246.38 (18) Å^3^	0.12 × 0.12 × 0.10 mm

### Data collection

**Table:**

Bruker APEXII CCD area-detector diffractometer	4402 reflections with *I* > 2σ(*I*)
Radiation source: fine-focus sealed tube	*R*_int_ = 0.026
φ and ω scans	θ_max_ = 26.4°, θ_min_ = 3.7°
Absorption correction: multi-scan (SADABS; Sheldrick, 2015)	*h* = −11→11
*T*_min_ = 0.981, *T*_max_ = 0.995	*k* = −13→13
22549 measured reflections	*l* = −17→18
5093 independent reflections	

### Refinement

**Table:**

Refinement on *F*^2^	0 restraints
Least-squares matrix: full	Hydrogen site location: inferred from neighbouring sites
*R*[*F*^2^ > 2σ(*F*^2^)] = 0.035	H-atom parameters constrained
*wR*(*F*^2^) = 0.104	*w* = 1/[σ^2^(*F*_o_^2^) + (0.0589*P*)^2^ + 0.2438*P*] where *P* = (*F*_o_^2^ + 2*F*_c_^2^)/3
*S* = 1.04	(Δ/σ)_max_ = 0.001
5093 reflections	Δρ_max_ = 0.22 e Å^−^^3^
343 parameters	Δρ_min_ = −0.19 e Å^−^^3^

### Special details

**Table:**

Geometry. All esds (except the esd in the dihedral angle between two l.s. planes) are estimated using the full covariance matrix. The cell esds are taken into account individually in the estimation of esds in distances, angles and torsion angles; correlations between esds in cell parameters are only used when they are defined by crystal symmetry. An approximate (isotropic) treatment of cell esds is used for estimating esds involving l.s. planes.

### Fractional atomic coordinates and isotropic or equivalent isotropic displacement parameters (Å^2^)

**Table:**

	*x*	*y*	*z*	*U*_iso_*/*U*_eq_	
N1	1.02807 (12)	0.88812 (10)	0.68529 (7)	0.0324 (2)	
C1	0.94092 (13)	0.76789 (11)	0.65188 (7)	0.0229 (2)	
C2	0.83242 (12)	0.61531 (11)	0.61689 (7)	0.0196 (2)	
C3	0.77520 (11)	0.54507 (11)	0.52005 (7)	0.0191 (2)	
C4	0.80379 (11)	0.62187 (11)	0.44699 (7)	0.0200 (2)	
C5	0.79817 (13)	0.75520 (12)	0.46234 (8)	0.0241 (2)	
H5	0.7715	0.7965	0.5187	0.029*	
C6	0.83202 (14)	0.82631 (12)	0.39420 (8)	0.0279 (2)	
H6	0.8284	0.9150	0.4054	0.033*	
C7	0.87130 (13)	0.76576 (12)	0.30952 (8)	0.0273 (2)	
H7	0.8958	0.8144	0.2645	0.033*	
C8	0.87392 (13)	0.63244 (12)	0.29233 (8)	0.0266 (2)	
H8	0.8992	0.5912	0.2353	0.032*	
C9	0.83892 (12)	0.56010 (11)	0.35990 (7)	0.0228 (2)	
H9	0.8388	0.4698	0.3472	0.027*	
C10	0.68717 (12)	0.38450 (11)	0.48036 (7)	0.0208 (2)	
C11	0.54277 (13)	0.31868 (12)	0.41204 (7)	0.0265 (2)	
H11	0.4995	0.3752	0.3924	0.032*	
C12	0.46275 (15)	0.16898 (13)	0.37298 (8)	0.0369 (3)	
H12	0.3654	0.1259	0.3282	0.044*	
C13	0.52712 (17)	0.08421 (13)	0.40034 (9)	0.0420 (3)	
H13	0.4735	−0.0159	0.3740	0.050*	
C14	0.67154 (18)	0.14849 (13)	0.46697 (10)	0.0402 (3)	
H14	0.7157	0.0914	0.4849	0.048*	
C15	0.75108 (14)	0.29761 (12)	0.50735 (9)	0.0297 (3)	
H15	0.8476	0.3399	0.5527	0.036*	
C16	0.79833 (12)	0.55102 (10)	0.69650 (7)	0.0191 (2)	
C17	0.64459 (12)	0.44808 (12)	0.69409 (8)	0.0243 (2)	
H17	0.5606	0.4181	0.6418	0.029*	
C18	0.61664 (12)	0.39052 (12)	0.76912 (8)	0.0251 (2)	
H18	0.5137	0.3226	0.7666	0.030*	
C19	0.74037 (12)	0.43273 (11)	0.84834 (7)	0.0197 (2)	
C20	0.89266 (12)	0.53771 (11)	0.85158 (7)	0.0215 (2)	
H20	0.9763	0.5683	0.9042	0.026*	
C21	0.92078 (12)	0.59701 (11)	0.77726 (7)	0.0207 (2)	
H21	1.0225	0.6683	0.7813	0.025*	
C22	0.71039 (11)	0.36891 (11)	0.92838 (7)	0.0197 (2)	
C23	0.65220 (12)	0.42907 (11)	1.00476 (8)	0.0228 (2)	
H23	0.6337	0.5090	1.0052	0.027*	
C24	0.62136 (12)	0.37253 (11)	1.07996 (7)	0.0239 (2)	
H24	0.5815	0.4142	1.1296	0.029*	
C25	0.64954 (11)	0.25348 (11)	1.08188 (7)	0.0205 (2)	
C26	0.62014 (12)	0.19169 (12)	1.15898 (7)	0.0254 (2)	
H26	0.5804	0.2318	1.2094	0.031*	
C27	0.64880 (13)	0.07757 (12)	1.15974 (8)	0.0275 (2)	
H27	0.6296	0.0412	1.2110	0.033*	
C28	0.70848 (12)	0.01052 (11)	1.08316 (8)	0.0242 (2)	
C29	0.73803 (13)	−0.10905 (12)	1.08179 (9)	0.0306 (3)	
H29	0.7200	−0.1470	1.1324	0.037*	
C30	0.79388 (14)	−0.17187 (12)	1.00585 (9)	0.0327 (3)	
H30	0.8124	−0.2516	1.0061	0.039*	
C31	0.82237 (13)	−0.11705 (12)	0.92967 (9)	0.0287 (2)	
H31	0.8589	−0.1607	0.8790	0.034*	
C32	0.79652 (12)	0.00371 (11)	0.92856 (8)	0.0227 (2)	
C33	0.83002 (12)	0.06669 (11)	0.85275 (8)	0.0233 (2)	
H33	0.8716	0.0273	0.8032	0.028*	
C34	0.80244 (12)	0.18199 (11)	0.85180 (7)	0.0214 (2)	
H34	0.8242	0.2193	0.8011	0.026*	
C35	0.74010 (11)	0.24854 (10)	0.92750 (7)	0.0182 (2)	
C36	0.70957 (11)	0.19041 (11)	1.00497 (7)	0.0187 (2)	
C37	0.73828 (11)	0.06826 (11)	1.00549 (7)	0.0205 (2)	

### Atomic displacement parameters (Å^2^)

**Table:**

	*U*^11^	*U*^22^	*U*^33^	*U*^12^	*U*^13^	*U*^23^
N1	0.0421 (6)	0.0230 (5)	0.0248 (5)	0.0069 (4)	0.0025 (4)	0.0092 (4)
C1	0.0288 (5)	0.0241 (6)	0.0177 (5)	0.0112 (5)	0.0060 (4)	0.0100 (4)
C2	0.0196 (5)	0.0189 (5)	0.0221 (5)	0.0087 (4)	0.0052 (4)	0.0087 (4)
C3	0.0165 (4)	0.0208 (5)	0.0224 (5)	0.0093 (4)	0.0049 (4)	0.0085 (4)
C4	0.0171 (4)	0.0214 (5)	0.0199 (5)	0.0067 (4)	0.0027 (4)	0.0073 (4)
C5	0.0273 (5)	0.0268 (5)	0.0221 (5)	0.0139 (4)	0.0077 (4)	0.0097 (4)
C6	0.0331 (6)	0.0272 (6)	0.0292 (6)	0.0151 (5)	0.0082 (5)	0.0144 (5)
C7	0.0278 (5)	0.0326 (6)	0.0236 (5)	0.0101 (5)	0.0072 (4)	0.0163 (5)
C8	0.0252 (5)	0.0317 (6)	0.0202 (5)	0.0095 (5)	0.0067 (4)	0.0078 (4)
C9	0.0220 (5)	0.0221 (5)	0.0225 (5)	0.0084 (4)	0.0044 (4)	0.0062 (4)
C10	0.0214 (5)	0.0210 (5)	0.0201 (5)	0.0081 (4)	0.0083 (4)	0.0073 (4)
C11	0.0244 (5)	0.0308 (6)	0.0204 (5)	0.0081 (4)	0.0067 (4)	0.0076 (4)
C12	0.0323 (6)	0.0337 (6)	0.0232 (6)	−0.0020 (5)	0.0087 (5)	0.0006 (5)
C13	0.0559 (8)	0.0195 (6)	0.0365 (7)	0.0037 (6)	0.0234 (6)	0.0025 (5)
C14	0.0576 (8)	0.0267 (6)	0.0465 (7)	0.0232 (6)	0.0239 (6)	0.0157 (6)
C15	0.0318 (6)	0.0273 (6)	0.0342 (6)	0.0154 (5)	0.0088 (5)	0.0117 (5)
C16	0.0218 (5)	0.0177 (5)	0.0199 (5)	0.0099 (4)	0.0054 (4)	0.0072 (4)
C17	0.0193 (5)	0.0295 (6)	0.0234 (5)	0.0082 (4)	0.0008 (4)	0.0121 (4)
C18	0.0174 (5)	0.0274 (5)	0.0280 (5)	0.0049 (4)	0.0031 (4)	0.0133 (4)
C19	0.0214 (5)	0.0188 (5)	0.0212 (5)	0.0101 (4)	0.0053 (4)	0.0080 (4)
C20	0.0201 (5)	0.0218 (5)	0.0198 (5)	0.0070 (4)	0.0007 (4)	0.0067 (4)
C21	0.0192 (5)	0.0176 (5)	0.0227 (5)	0.0049 (4)	0.0045 (4)	0.0069 (4)
C22	0.0154 (4)	0.0203 (5)	0.0196 (5)	0.0042 (4)	0.0009 (4)	0.0071 (4)
C23	0.0213 (5)	0.0206 (5)	0.0260 (5)	0.0089 (4)	0.0048 (4)	0.0071 (4)
C24	0.0215 (5)	0.0254 (5)	0.0201 (5)	0.0078 (4)	0.0057 (4)	0.0034 (4)
C25	0.0153 (4)	0.0222 (5)	0.0170 (5)	0.0024 (4)	0.0008 (4)	0.0054 (4)
C26	0.0197 (5)	0.0317 (6)	0.0163 (5)	0.0033 (4)	0.0028 (4)	0.0070 (4)
C27	0.0211 (5)	0.0332 (6)	0.0207 (5)	0.0010 (4)	0.0005 (4)	0.0149 (4)
C28	0.0168 (5)	0.0236 (5)	0.0252 (5)	0.0010 (4)	−0.0032 (4)	0.0113 (4)
C29	0.0230 (5)	0.0263 (6)	0.0361 (6)	0.0013 (4)	−0.0045 (5)	0.0180 (5)
C30	0.0257 (6)	0.0203 (5)	0.0477 (7)	0.0062 (4)	−0.0038 (5)	0.0137 (5)
C31	0.0230 (5)	0.0202 (5)	0.0377 (6)	0.0072 (4)	0.0003 (4)	0.0062 (5)
C32	0.0169 (5)	0.0194 (5)	0.0259 (5)	0.0046 (4)	−0.0015 (4)	0.0053 (4)
C33	0.0207 (5)	0.0239 (5)	0.0216 (5)	0.0087 (4)	0.0038 (4)	0.0033 (4)
C34	0.0206 (5)	0.0243 (5)	0.0169 (5)	0.0070 (4)	0.0039 (4)	0.0072 (4)
C35	0.0147 (4)	0.0185 (5)	0.0170 (5)	0.0037 (4)	0.0007 (4)	0.0050 (4)
C36	0.0142 (4)	0.0191 (5)	0.0169 (5)	0.0025 (4)	−0.0005 (3)	0.0051 (4)
C37	0.0147 (5)	0.0192 (5)	0.0212 (5)	0.0021 (4)	−0.0027 (4)	0.0069 (4)

### Geometric parameters (Å, º)

**Table:**

N1—C1	1.1479 (14)	C19—C20	1.3953 (14)
C1—C2	1.4482 (14)	C19—C22	1.4945 (13)
C2—C3	1.3623 (14)	C20—C21	1.3881 (14)
C2—C16	1.4947 (13)	C20—H20	0.9300
C3—C4	1.4889 (13)	C21—H21	0.9300
C3—C10	1.4911 (14)	C22—C23	1.3950 (14)
C4—C5	1.4002 (15)	C22—C35	1.4094 (14)
C4—C9	1.4006 (14)	C23—C24	1.3853 (15)
C5—C6	1.3878 (14)	C23—H23	0.9300
C5—H5	0.9300	C24—C25	1.3971 (15)
C6—C7	1.3876 (16)	C24—H24	0.9300
C6—H6	0.9300	C25—C36	1.4240 (14)
C7—C8	1.3852 (16)	C25—C26	1.4419 (14)
C7—H7	0.9300	C26—C27	1.3438 (17)
C8—C9	1.3898 (15)	C26—H26	0.9300
C8—H8	0.9300	C27—C28	1.4369 (17)
C9—H9	0.9300	C27—H27	0.9300
C10—C11	1.3926 (15)	C28—C29	1.3986 (16)
C10—C15	1.3938 (15)	C28—C37	1.4251 (14)
C11—C12	1.3909 (16)	C29—C30	1.3884 (19)
C11—H11	0.9300	C29—H29	0.9300
C12—C13	1.378 (2)	C30—C31	1.3864 (17)
C12—H12	0.9300	C30—H30	0.9300
C13—C14	1.382 (2)	C31—C32	1.4007 (15)
C13—H13	0.9300	C31—H31	0.9300
C14—C15	1.3871 (17)	C32—C37	1.4203 (15)
C14—H14	0.9300	C32—C33	1.4352 (15)
C15—H15	0.9300	C33—C34	1.3514 (15)
C16—C21	1.3967 (14)	C33—H33	0.9300
C16—C17	1.3994 (14)	C34—C35	1.4417 (14)
C17—C18	1.3868 (14)	C34—H34	0.9300
C17—H17	0.9300	C35—C36	1.4254 (13)
C18—C19	1.3967 (14)	C36—C37	1.4275 (15)
C18—H18	0.9300		
			
N1—C1—C2	175.73 (11)	C21—C20—C19	120.78 (9)
C3—C2—C1	120.14 (9)	C21—C20—H20	119.6
C3—C2—C16	126.86 (9)	C19—C20—H20	119.6
C1—C2—C16	112.98 (8)	C20—C21—C16	120.86 (9)
C2—C3—C4	122.58 (9)	C20—C21—H21	119.6
C2—C3—C10	121.61 (9)	C16—C21—H21	119.6
C4—C3—C10	115.73 (8)	C23—C22—C35	119.64 (9)
C5—C4—C9	118.34 (9)	C23—C22—C19	119.62 (9)
C5—C4—C3	122.32 (9)	C35—C22—C19	120.74 (9)
C9—C4—C3	119.33 (9)	C24—C23—C22	121.53 (10)
C6—C5—C4	120.63 (10)	C24—C23—H23	119.2
C6—C5—H5	119.7	C22—C23—H23	119.2
C4—C5—H5	119.7	C23—C24—C25	120.60 (9)
C7—C6—C5	120.35 (10)	C23—C24—H24	119.7
C7—C6—H6	119.8	C25—C24—H24	119.7
C5—C6—H6	119.8	C24—C25—C36	118.98 (9)
C8—C7—C6	119.69 (10)	C24—C25—C26	122.48 (10)
C8—C7—H7	120.2	C36—C25—C26	118.54 (10)
C6—C7—H7	120.2	C27—C26—C25	121.57 (10)
C7—C8—C9	120.26 (10)	C27—C26—H26	119.2
C7—C8—H8	119.9	C25—C26—H26	119.2
C9—C8—H8	119.9	C26—C27—C28	121.50 (9)
C8—C9—C4	120.67 (10)	C26—C27—H27	119.3
C8—C9—H9	119.7	C28—C27—H27	119.3
C4—C9—H9	119.7	C29—C28—C37	118.84 (10)
C11—C10—C15	118.72 (10)	C29—C28—C27	122.65 (10)
C11—C10—C3	120.39 (9)	C37—C28—C27	118.52 (10)
C15—C10—C3	120.83 (9)	C30—C29—C28	120.91 (10)
C12—C11—C10	120.46 (11)	C30—C29—H29	119.5
C12—C11—H11	119.8	C28—C29—H29	119.5
C10—C11—H11	119.8	C31—C30—C29	120.71 (10)
C13—C12—C11	120.26 (12)	C31—C30—H30	119.6
C13—C12—H12	119.9	C29—C30—H30	119.6
C11—C12—H12	119.9	C30—C31—C32	120.40 (11)
C12—C13—C14	119.74 (11)	C30—C31—H31	119.8
C12—C13—H13	120.1	C32—C31—H31	119.8
C14—C13—H13	120.1	C31—C32—C37	119.40 (10)
C13—C14—C15	120.42 (12)	C31—C32—C33	122.04 (10)
C13—C14—H14	119.8	C37—C32—C33	118.55 (9)
C15—C14—H14	119.8	C34—C33—C32	121.47 (9)
C14—C15—C10	120.38 (11)	C34—C33—H33	119.3
C14—C15—H15	119.8	C32—C33—H33	119.3
C10—C15—H15	119.8	C33—C34—C35	121.57 (9)
C21—C16—C17	118.43 (9)	C33—C34—H34	119.2
C21—C16—C2	119.78 (9)	C35—C34—H34	119.2
C17—C16—C2	121.76 (9)	C22—C35—C36	119.10 (9)
C18—C17—C16	120.43 (9)	C22—C35—C34	122.76 (9)
C18—C17—H17	119.8	C36—C35—C34	118.13 (9)
C16—C17—H17	119.8	C25—C36—C35	120.14 (9)
C17—C18—C19	121.18 (9)	C25—C36—C37	119.75 (9)
C17—C18—H18	119.4	C35—C36—C37	120.11 (9)
C19—C18—H18	119.4	C32—C37—C28	119.74 (10)
C20—C19—C18	118.26 (9)	C32—C37—C36	120.13 (9)
C20—C19—C22	120.70 (9)	C28—C37—C36	120.13 (10)
C18—C19—C22	121.04 (9)		
			
C1—C2—C3—C4	−7.36 (15)	C35—C22—C23—C24	−0.53 (15)
C16—C2—C3—C4	173.99 (9)	C19—C22—C23—C24	179.41 (9)
C1—C2—C3—C10	169.22 (9)	C22—C23—C24—C25	0.67 (15)
C16—C2—C3—C10	−9.43 (15)	C23—C24—C25—C36	−0.39 (15)
C2—C3—C4—C5	−39.58 (14)	C23—C24—C25—C26	179.60 (9)
C10—C3—C4—C5	143.65 (10)	C24—C25—C26—C27	−179.66 (10)
C2—C3—C4—C9	139.89 (10)	C36—C25—C26—C27	0.33 (15)
C10—C3—C4—C9	−36.88 (12)	C25—C26—C27—C28	−0.70 (16)
C9—C4—C5—C6	−2.14 (15)	C26—C27—C28—C29	−179.46 (10)
C3—C4—C5—C6	177.33 (9)	C26—C27—C28—C37	0.40 (15)
C4—C5—C6—C7	0.29 (16)	C37—C28—C29—C30	−0.64 (15)
C5—C6—C7—C8	1.12 (17)	C27—C28—C29—C30	179.22 (10)
C6—C7—C8—C9	−0.62 (16)	C28—C29—C30—C31	0.28 (16)
C7—C8—C9—C4	−1.29 (15)	C29—C30—C31—C32	0.57 (16)
C5—C4—C9—C8	2.65 (15)	C30—C31—C32—C37	−1.02 (15)
C3—C4—C9—C8	−176.85 (9)	C30—C31—C32—C33	177.76 (9)
C2—C3—C10—C11	132.68 (10)	C31—C32—C33—C34	179.06 (10)
C4—C3—C10—C11	−50.52 (12)	C37—C32—C33—C34	−2.15 (15)
C2—C3—C10—C15	−50.29 (14)	C32—C33—C34—C35	0.84 (15)
C4—C3—C10—C15	126.51 (10)	C23—C22—C35—C36	0.13 (14)
C15—C10—C11—C12	1.15 (15)	C19—C22—C35—C36	−179.81 (8)
C3—C10—C11—C12	178.24 (9)	C23—C22—C35—C34	−179.06 (9)
C10—C11—C12—C13	−1.11 (16)	C19—C22—C35—C34	1.00 (14)
C11—C12—C13—C14	0.13 (18)	C33—C34—C35—C22	179.93 (9)
C12—C13—C14—C15	0.79 (18)	C33—C34—C35—C36	0.73 (14)
C13—C14—C15—C10	−0.73 (18)	C24—C25—C36—C35	−0.01 (14)
C11—C10—C15—C14	−0.24 (16)	C26—C25—C36—C35	180.00 (8)
C3—C10—C15—C14	−177.31 (10)	C24—C25—C36—C37	−179.68 (9)
C3—C2—C16—C21	141.70 (11)	C26—C25—C36—C37	0.33 (14)
C1—C2—C16—C21	−37.03 (13)	C22—C35—C36—C25	0.13 (14)
C3—C2—C16—C17	−40.26 (15)	C34—C35—C36—C25	179.36 (8)
C1—C2—C16—C17	141.01 (10)	C22—C35—C36—C37	179.80 (8)
C21—C16—C17—C18	−1.85 (16)	C34—C35—C36—C37	−0.97 (14)
C2—C16—C17—C18	−179.92 (10)	C31—C32—C37—C28	0.64 (14)
C16—C17—C18—C19	−0.35 (17)	C33—C32—C37—C28	−178.18 (9)
C17—C18—C19—C20	1.73 (16)	C31—C32—C37—C36	−179.31 (9)
C17—C18—C19—C22	−179.10 (10)	C33—C32—C37—C36	1.87 (14)
C18—C19—C20—C21	−0.91 (15)	C29—C28—C37—C32	0.18 (14)
C22—C19—C20—C21	179.93 (9)	C27—C28—C37—C32	−179.68 (9)
C19—C20—C21—C16	−1.31 (16)	C29—C28—C37—C36	−179.88 (9)
C17—C16—C21—C20	2.68 (15)	C27—C28—C37—C36	0.26 (14)
C2—C16—C21—C20	−179.22 (9)	C25—C36—C37—C32	179.33 (8)
C20—C19—C22—C23	93.06 (12)	C35—C36—C37—C32	−0.34 (14)
C18—C19—C22—C23	−86.08 (13)	C25—C36—C37—C28	−0.61 (14)
C20—C19—C22—C35	−87.00 (12)	C35—C36—C37—C28	179.71 (8)
C18—C19—C22—C35	93.85 (12)		

### Hydrogen-bond geometry (Å, º)

**Table:**

*D*—H···*A*	*D*—H	H···*A*	*D*···*A*	*D*—H···*A*
C6—H6···N1^i^	0.93	2.73	3.3563 (17)	125

Symmetry code: (i) −*x*+2, −*y*+2, −*z*+1.
